# Global, regional, and national burden of self-harm among adolescents aged 10-24 years from 1990 to 2021, temporal trends, health inequities and projection to 2041

**DOI:** 10.3389/fpsyt.2025.1564537

**Published:** 2025-03-17

**Authors:** Jiang Tan, Yanping Shu, Qing Li, Lifan Liang, Yu Zhang, Jiyuan Zhang, Gang Wu, Yu Luo

**Affiliations:** ^1^ School of Psychology, Guizhou Normal University, Guiyang, China; ^2^ Key Laboratory of Brain Function and Brain Disease Prevention and Treatment of Guizhou Province, Guiyang, China; ^3^ Department of Psychiatry, The Second People’s Hospital of Guizhou Province, Guiyang, China; ^4^ The Second Clinical Medical College, Guizhou University of Traditional Chinese Medicine, Guiyang, China; ^5^ College of Inland Open Economics, Guizhou University of Commerce, Guiyang, China

**Keywords:** self-harm, adolescents, global burden of disease, health inequities, temporal trend, Bayesian age-period-cohort, socio-demographic index

## Abstract

**Background:**

Self-harm ranks as the third leading cause of disability-adjusted life years (DALYs) among adolescents globally, imposing substantial disease and economic burdens. Comprehensive analyses of global temporal trends, health inequities, and future projections are crucial for developing effective public health policies and interventions.

**Methods:**

This study analyzed the global, regional, and national age-standardized incidence, mortality, and DALYs for self-harm among adolescents using data from the Global Burden of Disease (GBD) 2021 database. Significant disease burdens and temporal trends were assessed. Projections and evaluations employed a combination of health inequities analysis, age-period-cohort (APC) analysis, socio-demographic index (SDI) analysis, Joinpoint regression analysis, and Bayesian APC modeling.

**Results:**

The global burden of self-harm among adolescents demonstrated an overall downward trend. However, in 2021, the burden increased with age and is projected to decline further by 2041. Joinpoint regression analysis revealed a generally decreasing temporal trend, although some regions exhibited stable or slightly increasing trends. Significant regional and national heterogeneities were identified. The High SDI region showed a slight upward trend in incidence, Southern Latin America experienced the largest increase, and the Middle SDI region showed the largest decrease. Conversely, East Asia demonstrated the most significant reductions in both incidence and mortality. Age effects were most pronounced in Low-middle SDI regions, while period and cohort effects exhibited greater fluctuations in High SDI regions. Notably, SDI analysis revealed a positive, fluctuating nonlinear relationship with age-standardized DALYs (*r* = 0.324, *P* < 0.001). Gender and regional disparities were also significant. Male adolescents in Middle and High SDI regions bore a higher burden of mortality, whereas female adolescents in Low SDI regions experienced a disproportionately high incidence. Adolescents aged 15-24 carried the greatest burden, with females exhibiting a higher incidence and males experiencing higher mortality rates.

**Conclusion:**

Despite an overall decline, significant gender and regional disparities persist. Male adolescents in higher SDI regions and females in lower SDI regions are particularly vulnerable. These findings underscore the need for targeted interventions addressing gender and regional inequalities, optimizing healthcare resource allocation, improving health education, and reducing the socioeconomic costs associated with self-harm in adolescents.

## 1 Introduction

Self-harm is a complex and significant public health issue that has garnered widespread global concern (1, 2). It is typically defined as intentional self-poisoning or self-injury regardless of its apparent purpose and encompasses behaviors such as drug overdose, ingestion of harmful substances, scratching, cutting, burning, or punching (3, 4). Self-harm is commonly classified into two categories: self-harm with suicidal intent (attempted suicide) and self-harm without suicidal intent (non-suicidal self-injury, NSSI) (5–7). Notably, the latter has been recognized in the Diagnostic and Statistical Manual of Mental Disorders (5th edition) as a condition warranting further research (8). Individuals diagnosed with mental health disorders, such as depression and anxiety, are at a significantly higher risk of engaging in self-harming behaviors (9). Furthermore, the high global incidence of self-harm may contribute to an increased risk of premature death (10, 11). Estimates suggest that at least 14 million incidents of self-harm occur worldwide each year, equating to approximately 60 out of every 100,000 people engaging in such behaviors annually (12). In 2021, there were an estimated 5.49 million new cases of self-harm globally, and projections indicate that this number may rise to 10.55 million by 2040 (13). Research indicates that self-harm occurs across all age groups, with lifetime prevalence rates estimated at approximately 3% in adults and 14% in children and adolescents (14, 15). However, notable gender differences exist in self-harm prevalence, with females exhibiting higher rates than males. Self-harm is also a leading cause of disability-adjusted life years (DALYs) among adolescents aged 10-24, ranking as the third-highest contributor to DALYs in this population globally (12, 16).

Although self-harm can occur at any age, it typically begins during adolescence, when prevalence rates are particularly high (10, 17). Adolescents with a history of self-harm are often underrepresented in prevalence estimates due to factors such as stigma, lack of access to care, and low visibility. This is especially true in low- and middle-income countries, where resources for mental health care are often unevenly distributed (18, 19). According to the Global Burden of Disease Study 2019 (GBD 2019), the global economic cost of self-harm among individuals under the age of 24 is substantial. The 34 million DALYs lost to self-harm in 2019 were valued at $639 billion globally (12). Of these costs, 81% were incurred in countries with low or Low-middle socio-demographic index (SDI) classifications. Notably, individuals under 25 years of age accounted for 25% of the global economic burden; in countries with Low or Low-middle SDI classifications, this share increased to over 33% (12).

Self-harm is characterized by a high rate of recurrence. Statistically, the annual recurrence rate for non-fatal self-harm is 16.3%, with one in three individuals engaging in repeat self-harm within as little as one month (20). A well-documented link also exists between self-harm and suicide, with 1.6% of individuals who self-harm dying by suicide within one year, and 6% dying by suicide in the subsequent years after seeking help from institutions such as hospitals (20, 21). Adolescent self-harm is closely linked to youth suicide, which remains a leading cause of mortality in this demographic. International initiatives such as the WHO Mental Health Action Plan and national suicide prevention policies emphasize early intervention strategies targeting self-harm behaviors as a critical component of suicide prevention (22). Expanding adolescent mental health services, including school-based interventions and digital mental health platforms, has been recognized as an essential public health measure to mitigate the burden of self-harm (23). Despite this strong association, self-harm tends to receive less attention and monitoring compared to suicide, though the global disease and economic burden it imposes cannot be overlooked.

A comprehensive analysis of temporal trends in the burden of self-harm among adolescents worldwide is essential to better understand its epidemiology, track progress in its management, and identify priorities for future interventions. Importantly, temporal trends are often influenced by multiple dimensions. For any given population, the risk of disease prevalence is shaped not only by biological age but also by the specific time period. This means that disease prevalence can be further analyzed through the lens of age, period, and birth cohort effects. Although global declines in both the incidence of self-harm and average annual suicide rates have been observed, the relative influence of these multidimensional time trends remains understudied. Moreover, a significant gap exists in the reporting of self-harm incidence trends among adolescents, as well as its relationship to temporal trends, across many countries, including those classified as low-, middle-, and high-income. The GBD 2021 study, which incorporates the latest epidemiological data and employs advanced statistical methods, provides a valuable opportunity to generate population-level health indicators and analyze temporal trends in disease burden from a global perspective. In this study, we extracted data from GBD 2021 to examine the burden of disease, temporal trends, health inequities, and projections of self-harm at global, regional, and national levels for the period 1990-2021. We focus on a cohort of adolescents aged 10-24 years because this broad definition of age more accurately reflects the expansion of biological growth and social role transitions that help to adapt to the changes and developments of modern society (24). The 10-24 age range is increasingly recognized as a crucial period for tracking self-harm trends, as it encompasses key developmental transitions, including neurocognitive maturation, identity formation, and shifts in social roles. This broader definition aligns with contemporary adolescent health frameworks and facilitates more comprehensive intervention strategies tailored to emerging adulthood (24). This definition is essential for the formulation of policies and the expansion of social services and concerns that are responsive to the developmental needs of adolescents.

## 2 Methods

### 2.1 GBD data overview

The GBD 2021 study, a comprehensive and up-to-date epidemiological analysis conducted by the Institute for Health Metrics and Evaluation (IHME), assessed the health impacts of 371 diseases, injuries, and risk factors across 204 countries and territories (25). It incorporated a wide range of health data sources, including censuses, household surveys, vital records, disease registries, healthcare utilization data, ambient air quality data, satellite imagery, and disease reports (26). The GBD study systematically addresses missing data through advanced statistical modeling rather than simple exclusion. Specifically, it employs spatiotemporal Gaussian process regression (ST-GPR) and cause-of-death ensemble modeling (CODEm) to estimate self-harm burden in regions where direct data is sparse or unavailable. These models enable smoothing across age, time, and location in regions with incomplete datasets, allowing for bias correction and data adjustment for further modeling. Additionally, discrepancies in self-harm reporting (i.e., regional inconsistencies) arise due to variations in healthcare infrastructure, surveillance systems, and classification standards across countries. To mitigate these discrepancies, GBD standardizes definitions and applies data adjustment techniques to correct for systematic underreporting or misclassification. Additionally, Bayesian meta-regression models, such as DisMod-MR, are used to synthesize data across regions while accounting for potential biases. The DisMod-MR tool synthesizes data from different regions and sources within a statistically probabilistic framework, generating a weighted average to account for heterogeneity and improve comparability (27). The GBD study adhered strictly to the Guidelines for Accurate and Transparent Health Estimation Reporting (GATHER) to ensure methodological rigor and transparency (28).

### 2.2 Data collection and descriptive statistics

We obtained estimates of prevalence rate, incidence rate, mortality rate, and DALYs rate, along with their 95% uncertainty intervals (UIs), for self-harm among adolescents from the GBD 2021 database (https://vizhub.healthdata.org/gbd-results/). Data were extracted for 204 countries and territories, 21 super-regions, and 5 SDI regions globally, focusing on adolescents aged 10-24 years (subdivided into three age groups: 10-14, 15-19, and 20-24 years) for the period 1990-2021. The broad age range of 10-24 years was adopted as it effectively captures the biological, social, and neurocognitive development of this population (24).

Descriptive statistical analyses were performed for age-standardized incidence rates (ASIR), age-standardized mortality rates (ASMR), age-standardized DALYs rates (ASDR), along with their estimated annual percentage changes (EAPC) for the years 1990 and 2021. EAPC values were calculated using a least-squares linear regression model to provide a robust assessment of temporal trends over the study period (7). An EAPC greater than 0 denoted an increasing trend in the burden of self-harm, whereas an EAPC less than 0 signified a decreasing trend. An EAPC equal to or near 0 indicated a relatively stable burden of self-harm over time.

To explore regional distributions and differences in the global burden of self-harm among adolescents between 1990 and 2021, DALYs were utilized to generate global maps and conduct comparative analyses. Additionally, a population-based analysis was performed to examine the distribution of self-harm across different demographic groups, stratified by age and sex. Specifically, data were analyzed for male and female subgroups within each age category. The relationship between SDI and the global burden of adolescent self-harm was also investigated. The SDI is a composite indicator that reflects the socioeconomic, demographic, and developmental levels of countries and regions. It is derived from metrics such as gross domestic product (GDP) per capita, average educational attainment, and total fertility rate. Based on their SDI values in the GBD 2021 study, 204 countries and territories were categorized into five quintiles: Low, Low-middle, Middle, High-middle, and High SDI regions (7). The SDI ranges from 0 to 1, with higher values indicating greater socioeconomic and developmental levels. Data processing and visualization were performed using the dplyr, ggplot2, and rnaturalearthdata packages in R software (version 4.4.0). These tools facilitated descriptive analysis and graphical representation of global, regional, and national trends in the burden of self-harm among adolescents.

### 2.3 Advanced analysis

#### 2.3.1 Cross-country inequalities analysis

This study employed the slope index of inequality (SII), as defined by the World Health Organization (WHO), and the concentration index to assess absolute and relative inequalities, respectively, in the burden of self-harm among adolescents across countries, relative to the SDI (29, 30). The SII was calculated by regressing DALYs rates against the midpoint of the cumulative hierarchical range of populations ranked by SDI, which served as a relative position indicator. The concentration index was calculated by measuring the deviation of the Lorenz curve from the line of equality, where the Lorenz curve plots the cumulative proportion of DALYs against the cumulative proportion of the population ranked by SDI (31). To evaluate changes in health inequality, data from 204 countries and territories were analyzed, disaggregated by sex, over the period from 1990 to 2021.

#### 2.3.2 Age-period-cohort model analysis

The APC model was employed as an advanced methodological approach to disentangle overall and specific temporal trends. This model is particularly advantageous in studies of health and socioeconomic development, as it accounts for net and local drifts in disease burden (32). The APC model simultaneously estimated the effects of age, period, and birth cohort on temporal trends in the disease burden. The mathematical equation of the model is as follows:


$$Log\left(Y_{ijk}\right)=\mu +\alpha_{i}+\beta_{j}+\gamma_{k}+\epsilon_{ijk}$$

Where Y_ijk_ denotes the prevalence of self-harm among adolescents observed in the i-th age group, j-th period, and k-th birth cohort; μ represents the overall mean; the α_i_, β_j_, and γ_k_ signify the age, period, and cohort effects, respectively; and ϵ_ijk_ refers to the error term.

For the APC analysis, age groups and periods were divided into 5-year intervals to ensure that the age interval equaled the period interval (32). Accordingly, the 1990–2021 time span was partitioned into six 5-year periods: 1992-1996, 1997-2001, 2002-2006, 2007-2011, 2012-2016, and 2017-2021. Consequently, the analysis encompassed three partially overlapping birth cohorts spanning from 1978-1982 to 1997-2001. The APC model utilized net drift, representing the overall annual percentage change in prevalence, and local drift, quantifying the annual percentage change in prevalence for each age group, to analyze overall trends and age-specific changes in prevalence. Given the sensitivity of fit rates over 30 years to minor variations in drift values, the Wald χ^2^ test was employed to assess the significance of trend changes. Furthermore, the APC model provided insights into how different birth cohorts and periods were influenced by socioeconomic factors, offering crucial information about epidemiological transitions in the disease burden.

#### 2.3.3 Bayesian age-period-cohort model analysis

To extend the findings of the APC model, the BAPC model was utilized to project trends in the disease burden of adolescent self-harm over the next 20 years (2022-2041). The BAPC model incorporates hierarchical Bayesian priors, specifically random walk (RW) priors for age, period, and cohort effects, ensuring smooth transitions while addressing the covariance issues inherent in APC models. Additionally, the model utilizes Penalized Complexity (PC) priors within the Integrated Nested Laplace Approximation (INLA) algorithm to regularize estimates and prevent overfitting. These priors help maintain epidemiological plausibility while improving model stability. The mathematical equation for the BAPC model is:


$$Log\left(\lambda_{ij}\right)=\alpha +\mu_{i}+\beta_{j}+\gamma_{k}$$

Where λ_ij_ represents the prevalence, α is the intercept term, μi denotes the age effect, β_j_ denotes the period effect, and γ_k_ denotes the cohort effect. Projections using the BAPC model were implemented via the R packages BAPC and INLA (33).

#### 2.3.4 Joinpoint regression analysis

The Joinpoint regression model was employed to estimate the average annual percentage change (AAPC) and its corresponding 95% confidence interval (CI), facilitating the evaluation of significant variations in internal trends across distinct periods (34, 35). This method is recognized for its high sensitivity and precision in detecting significant changes in linear slope points within temporal trends. Subsequently, key turning points in the prevalence trend were identified, and the simplest model was constructed by connecting multiple linear segments on a logarithmic scale. Joinpoint regression analysis enabled the identification of periods characterized by significant increases or decreases in the burden of self-harm among adolescents, thereby providing a deeper understanding of changes in underlying risk factors and the effectiveness of public health interventions.

### 2.4 Instruments for statistical analysis

All data analyses and visualizations were conducted using R software (version 4.4.2) and JD_GBDR (V2.22, Jingding Medical Technology Co., Ltd.). Descriptive statistics are presented as means with 95% UIs or 95% CIs. Statistical significance was determined using a two-sided *P*-value threshold of 0.05.

## 3 Results

### 3.1 Global burden of self-harm among adolescents aged 10-24 years

**Table 1** presents the descriptive statistics for the incidence rate, mortality rate, DALYs rate, and EAPC for adolescents aged 10-24 years with self-harm at the global level, across 21 GBD regions, and within five SDI regions. In 2021, the global incidence rate, mortality rate, and DALYs rate for self-harm among adolescents aged 10-24 years per 100,000 population were 89.64 (95% UI: 57.88, 132.55), 5.93 (95% UI: 5.44, 6.51), and 420.14 (95% UI: 385.21, 462.19), respectively. Compared to 1990, all three metrics decreased by 31.47%, 38.10%, and 38.39%, respectively. Over the 31-year period from 1990 to 2021, the global incidence rate, mortality rate, and DALYs rate for adolescent self-harm demonstrated a declining trend, with EAPCs of -1.40 (95% CI: -1.49, -1.32), -1.78 (95% CI: -2.05, -1.51), and -1.79 (95% CI: -2.05, -1.53), respectively.

**Table 1:** Global incidence rate, mortality rate, DALYs rate, and EAPC for self-harm among adolescents aged 10-24 years from1990 to 2021.

Location	Incidence rate			Mortality rate			DALYs rate		
	ASIR 1990 (95% UI)	ASIR 2021 (95% UI)	EAPC (95% CI)	ASMR 1990 (95% UI)	ASMR 2021 (95% UI)	EAPC (95% CI)	ASDR 1990 (95% UI)	ASDR 2021 (95% UI)	EAPC (95% CI)
Global	130.81 (89.76, 186.50)	89.64 (57.88, 132.55)	-1.40 (-1.49, -1.32)	9.58 (7.88, 10.33)	5.93 (5.44, 6.51)	-1.78 (-2.05, -1.51)	681.99 (561.45, 735.81)	420.14 (385.21, 462.19)	-1.79 (-2.05, -1.53)
*Socio-demographic index*									
Low SDI	68.02 (46.28, 98.25)	54.23 (34.45, 81.34)	-1.01 (-1.11, -0.91)	6.65 (5.38, 8.09)	4.76 (4.05, 5.81)	-1.22 (-1.52, -0.92)	476.34 (386.43, 578.45)	339.43 (288.92, 414.02)	-1.23 (-1.53, -0.94)
Low-middle SDI	149.73 (102.55, 212.97)	98.87 (63.10, 147.12)	-1.48 (-1.63, -1.33)	13.40 (10.12, 15.22)	7.71 (6.71, 8.69)	-1.85 (-2.08, -1.62)	953.31 (722.40, 1081.41)	546.64 (476.24, 616.11)	-1.86 (-2.09, -1.63)
Middle SDI	123.70 (82.89, 179.06)	77.66 (49.08, 115.41)	-1.72 (-1.80, -1.64)	9.33 (6.95, 10.35)	5.15 (4.69, 5.66)	-2.02 (-2.24, -1.80)	664.87 (496.13, 735.56)	364.52 (332.18, 400.86)	-2.04 (-2.25, -1.84)
High-middle SDI	138.43 (93.93, 198.30)	95.61 (63.12, 138.58)	-1.32 (-1.52, -1.12)	8.29 (6.99, 9.07)	4.25 (3.91, 4.66)	-2.79 (-3.60, -1.98)	591.03 (498.81, 645.61)	300.21 (277.27, 328.90)	-2.81 (-3.60, -2.02)
High SDI	152.94 (111.11, 209.63)	155.91 (103.83, 225.41)	0.04 (-0.06, 0.14)	7.75 (7.56, 8.26)	7.11 (6.89, 7.33)	-0.27 (-1.32, 0.80)	545.61 (532.46, 581.99)	500.36 (484.50, 516.80)	-0.27 (-1.30, 0.77)
*GBD region*									
Central Asia	107.62 (89.14, 129.93)	145.82 (116.99, 182.44)	1.36 (1.20, 1.52)	7.69 (7.25, 8.13)	7.72 (6.89, 8.63)	0.14 (-0.65, 0.93)	550.59 (519.16, 581.59)	551.79 (492.18, 616.47)	0.13 (-0.64, 0.90)
Central Europe	119.47 (91.90, 153.52)	105.05 (72.29, 148.89)	-0.45 (-0.59, -0.31)	7.72 (7.41, 8.03)	4.89 (4.47, 5.18)	-1.46 (-2.86, -0.05)	546.89 (525.54, 568.81)	343.95 (315.21, 364.54)	-1.49 (-2.86, -0.11)
Eastern Europe	197.38 (131.56, 286.18)	179.99 (112.62, 270.90)	-0.23 (-0.67, 0.22)	12.45 (12.18, 12.72)	9.95 (8.93, 10.93)	-1.41 (-2.94, 0.15)	881.64 (861.94, 900.11)	698.79 (628.76, 766.07)	-1.43 (-2.94, 0.10)
Australasia	196.12 (157.84, 245.73)	181.47 (135.06, 237.18)	-0.49 (-0.60, -0.38)	11.84 (11.18, 12.54)	9.34 (8.61, 10.01)	-0.74 (-1.86, 0.40)	829.47 (782.93, 878.43)	656.84 (604.73, 704.51)	-0.73 (-1.83, 0.39)
High-income Asia Pacific	136.10 (96.02, 192.00)	166.87 (112.19, 240.64)	0.85 (0.52, 1.18)	6.75 (6.23, 8.86)	8.63 (8.01, 8.97)	0.89 (0.28, 1.49)	474.51 (438.04, 623.66)	604.93 (560.56, 628.47)	0.87 (0.28, 1.47)
High-income North America	206.84 (140.45, 295.83)	223.36 (144.41, 333.16)	0.27 (0.19, 0.34)	9.38 (9.16, 9.60)	9.86 (9.45, 10.27)	0.56 (-0.81, 1.96)	663.25 (647.58, 678.97)	695.91 (666.92, 724.62)	0.54 (-0.81, 1.91)
Southern Latin America	140.45 (112.11, 175.09)	208.61 (158.59, 269.27)	1.45 (1.23, 1.68)	8.00 (7.45, 8.54)	9.34 (8.49, 10.19)	0.96 (-0.28, 2.22)	563.67 (525.70, 601.34)	659.25 (599.91, 719.10)	0.96 (-0.25, 2.19)
Western Europe	112.33 (87.88, 142.82)	87.30 (61.24, 121.72)	-1.13 (-1.20, -1.05)	6.09 (5.93, 6.26)	3.68 (3.49, 3.84)	-1.88 (-2.21, -1.55)	426.25 (414.37, 437.97)	257.65 (244.81, 268.89)	-1.89 (-2.99, -0.79)
Andean Latin America	36.61 (27.91, 47.72)	44.91 (31.70, 62.00)	0.88 (0.66, 1.09)	4.65 (3.99, 5.50)	5.22 (4.26, 6.27)	0.63 (0.25, 1.02)	331.43 (284.30, 391.80)	372.11 (303.93, 446.42)	0.63 (0.26, 1.00)
Caribbean	64.22 (52.67, 77.53)	38.81 (26.54, 55.19)	-1.95 (-2.24, -1.66)	7.80 (7.13, 8.55)	4.26 (3.35, 5.33)	-1.74 (-2.23, -1.25)	549.57 (501.39, 602.29)	300.40 (236.47, 376.38)	-1.73 (-2.20, -1.25)
Central Latin America	32.87 (23.78, 45.05)	43.93 (28.36, 64.48)	1.11 (0.93, 1.28)	4.21 (4.01, 4.40)	6.73 (6.04, 7.44)	1.56 (0.55, 2.57)	298.36 (284.60, 312.08)	476.16 (426.78, 526.70)	1.54 (0.56, 2.53)
Tropical Latin America	26.98 (16.23, 41.53)	23.74 (14.32, 36.51)	-0.20 (-0.34, -0.05)	4.02 (3.80, 4.27)	5.66 (5.32, 6.05)	1.22 (0.17, 2.28)	282.74 (267.27, 300.30)	397.80 (373.42, 424.77)	1.21 (0.19, 2.24)
North Africa and Middle East	62.69 (43.72, 87.46)	57.42 (37.26, 85.80)	-0.44 (-0.54, -0.33)	4.64 (3.56, 5.40)	3.02 (2.45, 3.61)	-1.35 (-1.71, -0.98)	332.18 (256.06, 385.34)	214.74 (174.60, 256.37)	-1.38 (-1.73, -1.03)
East Asia	152.06 (97.91, 227.87)	69.53 (41.21, 108.29)	-2.82 (-3.02, -2.62)	10.64 (7.05, 12.36)	2.90 (2.40, 3.64)	-4.96 (-5.19, -4.74)	762.14 (506.89, 883.95)	205.69 (170.30, 257.81)	-4.99 (-5.20, -4.77)
Oceania	87.30 (64.62, 115.37)	74.88 (49.54, 108.22)	-0.75 (-0.84, -0.65)	7.27 (5.91, 8.80)	5.13 (4.11, 7.17)	-1.05 (-1.54, -0.56)	517.46 (421.82, 624.50)	365.40 (293.38, 510.45)	-1.06 (-1.54, -0.58)
South Asia	208.43 (140.44, 300.49)	134.99 (84.91, 203.07)	-1.62 (-1.79, -1.46)	17.42 (13.22, 19.89)	9.64 (8.44, 10.95)	-1.91 (-3.02, -0.78)	1239.74 (942.68, 1412.52)	683.53 (599.35, 775.80)	-1.89 (-2.23, -1.56)
Southeast Asia	81.03 (60.62, 106.68)	56.42 (36.62, 83.75)	-1.49 (-1.65, -1.34)	5.19 (4.43, 5.94)	2.99 (2.55, 3.49)	-2.18 (-2.89, -1.46)	369.02 (314.86, 421.35)	212.38 (181.92, 247.95)	-2.17 (-2.86, -1.48)
Central Sub-Saharan Africa	37.90 (26.17, 53.01)	35.97 (23.09, 53.34)	-0.31 (-0.35, -0.26)	4.78 (3.45, 6.73)	4.29 (2.96, 6.41)	-0.27 (-1.12, 0.59)	343.82 (249.30, 481.05)	305.55 (211.45, 455.96)	-0.30 (-1.13, 0.55)
Eastern Sub-Saharan Africa	40.58 (27.57, 58.00)	33.41 (21.06, 50.34)	-0.84 (-0.93, -0.76)	5.12 (4.10, 6.08)	3.89 (3.19, 4.91)	-1.16 (-1.94, -0.38)	368.40 (294.93, 436.49)	277.42 (227.99, 350.50)	-1.19 (-1.96, -0.42)
Southern Sub-Saharan Africa	84.32 (53.12, 127.45)	80.02 (47.98, 126.14)	-0.11 (-0.33, 0.12)	8.48 (6.63, 10.78)	10.52 (8.30, 12.70)	0.69 (-0.52, 1.92)	592.96 (465.22, 751.82)	736.11 (581.15, 887.96)	0.69 (-0.50, 1.90)
Western Sub-Saharan Africa	30.68 (20.57, 44.10)	31.46 (19.46, 47.79)	-0.06 (-0.12, 0.00)	2.86 (2.25, 3.50)	2.83 (2.04, 3.66)	-0.08 (-0.98, 0.83)	206.64 (163.33, 252.03)	202.57 (146.72, 262.13)	-0.10 (-0.99, 0.80)

DALYs, Disability-Adjusted Life-Years; ASIR, Age-Standardized Incidence Rates (Per 100,000 Population); ASMR, Age-Standardized Mortality Rates (Per 100,000 Population); ASDR, Age-Standardized DALYs Rates (Per 100,000 Population); SDI, Socio-Demographic Index; UI, Uncertainty Interval; EAPC, Estimated Annual Percentage Change.

The burden of adolescent self-harm across the five SDI regions exhibited distinct patterns. From Low to High SDI regions, the EAPC trajectories displayed an initial fluctuating decline followed by an upward trend, though the overall decline mirrored the global trends. In 2021, all SDI regions exhibited downward trends in incidence, mortality, and DALYs rates, except for a slight increase in the incidence rate in the High SDI region (EAPC: 0.04 [95% CI: -0.06, 0.14]), but these results were not statistically significant. Over the 31-year period, the Middle SDI region experienced the largest decline in incidence rate (AAPC: -1.72 [95% CI: -1.80, -1.64]). The most substantial decreases in mortality rate (AAPC: -2.79 [95% CI: -3.60, -1.98]) and DALYs rate (EAPC: -2.81 [95% CI: -3.60, -2.02]) were also observed in this region, with reductions nearly three times greater than those in other regions. In contrast, the High SDI region experienced only marginal declines in mortality rate (AAPC: -0.27 [95% CI: -1.32, 0.80]) and DALYs rate (AAPC: -0.27 [95% CI: -1.30, 0.77]), but these results were not statistically significant.

Among the 21 GBD regions, the three regions with the highest incidence rates of self-harm per 100,000 adolescents in 2021 were High-Income North America (223.36 [95% UI: 144.41, 333.16]), Southern Latin America (208.61 [95% UI: 158.59, 269.27]), and Australasia (181.47 [95% UI: 135.06, 237.18]). The three regions with the highest mortality rates were Southern Sub-Saharan Africa (10.52 [95% UI: 8.30, 12.70]), Eastern Europe (9.95 [95% UI: 8.93, 10.93]), and High-Income North America (9.86 [95% UI: 9.45, 10.27]). Similarly, the three regions with the highest DALYs rates were Southern Sub-Saharan Africa (736.11 [95% UI: 581.15, 887.96]), Eastern Europe (698.79 [95% UI: 628.76, 766.07]), and High-Income North America (695.91 [95% UI: 666.92, 724.62]).

The temporal trends of the disease burden for adolescent self-harm in the 21 regions were further evaluated using EAPC. Over the past 31 years, the global trends in EAPC for incidence, mortality, and DALYs rates varied significantly across regions. Notably, the largest increase in incidence rate was observed in Southern Latin America (EAPC: 1.45 [95% CI: 1.43, 1.68]), nearly five times higher than the smallest increase in High-Income North America (EAPC 0.27[95%CI 0.19, 0.34]). Conversely, the most significant decrease in incidence rate occurred in the East Asia region (EAPC: -2.82 [95% CI: -3.02, -2.62]), which was 47 times greater than the smallest decrease observed in Western Sub-Saharan Africa (EAPC: -0.06 [95% CI: -0.12, 0.00]). For mortality rate, the largest increase was observed in Central Latin America (EAPC: 1.56 [95% CI: 0.55, 2.57]), while the smallest increase occurred in Central Asia (EAPC: 0.14 [95% CI: -0.65, 0.93]). The largest decrease in mortality rate was found in East Asia (EAPC: -4.96 [95% CI: -5.19, -4.74]), which was 62 times greater than the smallest decrease in Western Sub-Saharan Africa (EAPC: -0.08 [95% CI: -0.98, 0.83]). A similar pattern was observed for DALYs rate, which closely mirrored the trends in mortality rate, further underscoring the regional disparities in the burden of adolescent self-harm.

**Figure 1:**
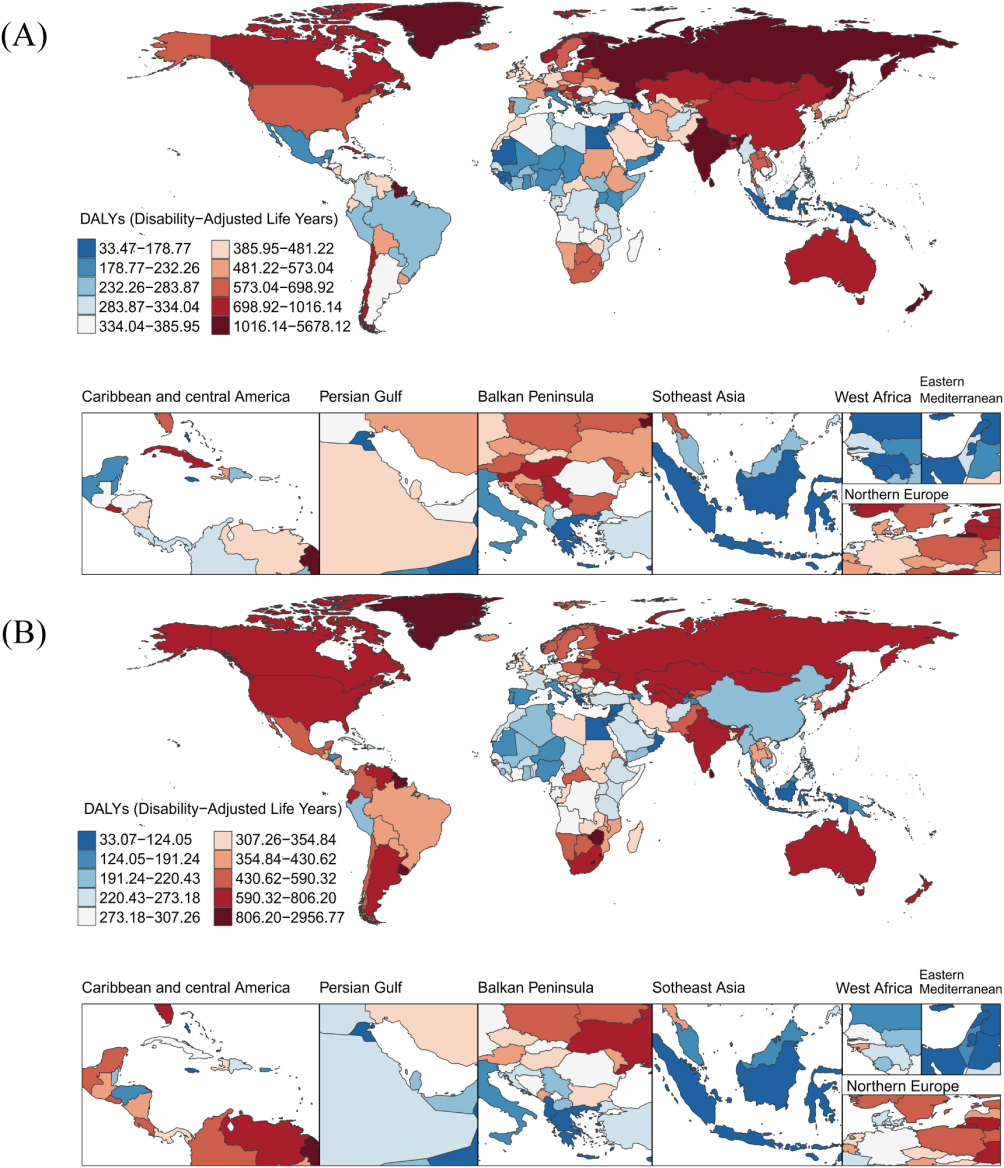
Global burden of disease for self-harm among adolescents in 204 countries and territories based on DALYs for 1990 **(A)** and 2021 **(B)**. DALYs, Disability-Adjusted Life-Years.

Among 204 countries and territories, **Table 1** illustrates the five countries with the highest ASDR for self-harm in 2021: Greenland (2927.50 [95% UI: 2164.01, 3525.99]), Nauru (2024.39 [95% UI: 1154.69, 2819.38]), Tokelau (1982.15 [95% UI: 1502.55, 2607.40]), Niue (1802.58 [95% UI: 1344.41, 2340.70]), and the Marshall Islands (1641.55 [95% UI: 980.21, 2296.53]) (**Figures 1A**, **Supplementary Table 1**). Greenland’s ASDR was 6.97 times higher than the global average. In contrast, Jamaica had the lowest ASDR in 2021 (33.41 [95% UI: 24.47, 45.17]). Over the 31-year period from 1990 to 2021, significant increases in ASDR were observed in Mexico and Zimbabwe, with rates more than tripling, while Seychelles and China showed the most rapid declines, decreasing nearly fourfold. Among individual countries, Lesotho (EAPC: 3.61 [95% CI: 2.30, 4.93]) and Zimbabwe (EAPC: 3.31 [95% CI: 2.27, 4.36]) exhibited the most pronounced increasing trends in ASDR. Conversely, China (EAPC: -5.11 [95% CI: -5.34, -4.89]) displayed the steepest decline, followed by Jordan (EAPC: -4.72 [95% CI: -5.37, -4.08]), Cuba (EAPC: -4.70 [95% CI: -5.51, -3.88]), Sri Lanka (EAPC: -4.65 [95% CI: -5.22, -4.08]), Luxembourg (EAPC: -4.61 [95% CI: -5.78, -3.43]), Serbia (EAPC: -4.36 [95% CI: -5.36, -3.35]), and Slovenia (EAPC: -4.00 [95% CI: -5.39, -2.60]). Notably, Pakistan exhibited no significant changes in ASDR over the 31 years, with an EAPC of 0%.

### 3.2 Age-gender-time trends in the burden of self-harm among adolescents

**Figure 2:**
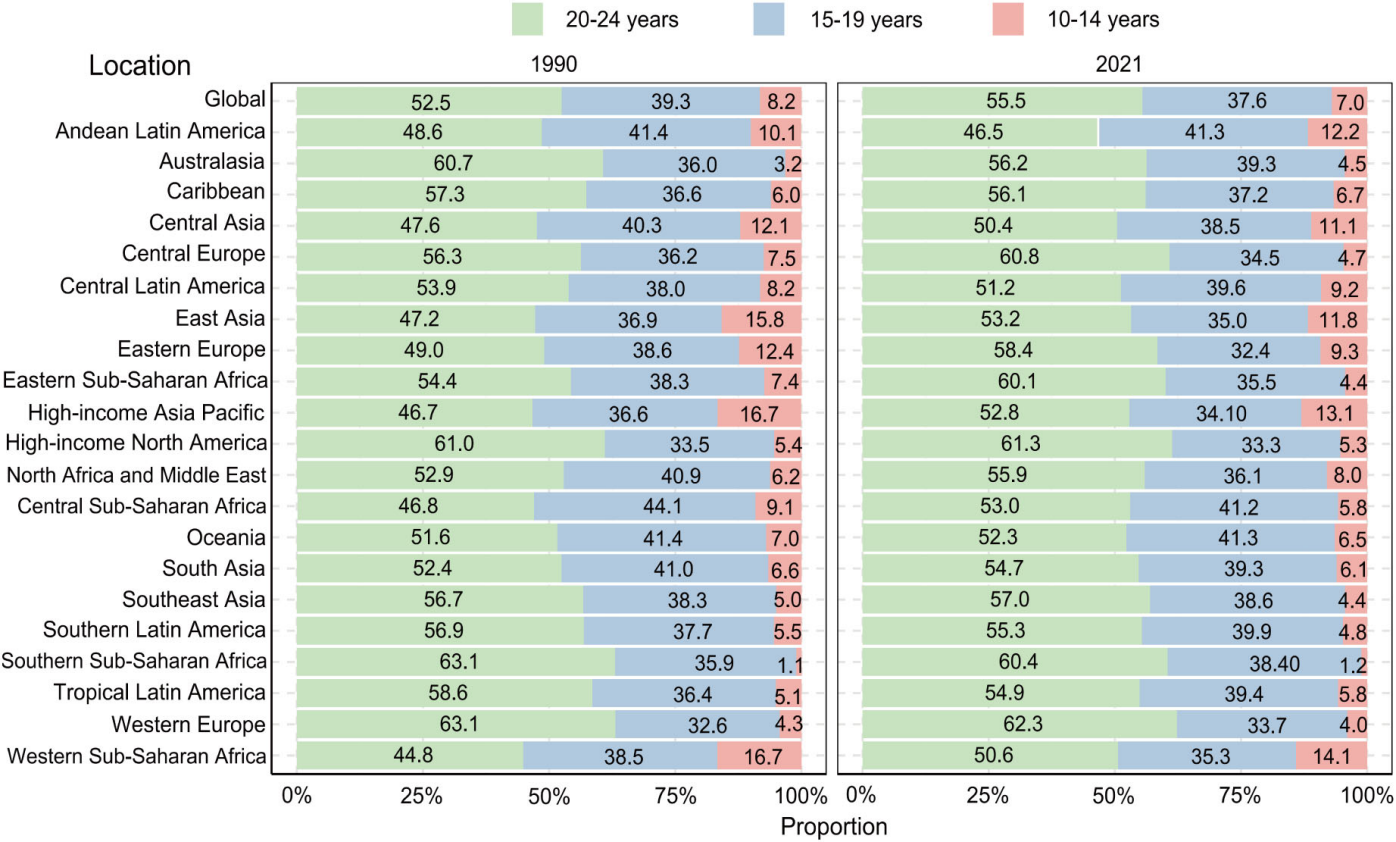
Prevalence proportion for self-harm among adolescents of different ages in 21 GBD super-regions and globally for 1990 and 2021.

Analysis of age-gender correlations revealed that the global prevalence and proportion of self-harm among adolescents increased with age, with similar trends observed for both males and females. However, the prevalence of self-harm was significantly higher among female adolescents compared to males across all age groups. The gender difference in prevalence was minimal among adolescents aged 10-14 years (**Figure 2**). Compared to 1990, the global prevalence of self-harm in 2021 generally decreased. However, there was a 3% increase in prevalence among adolescents aged 20-24 years. In 2021, the three regions with the highest prevalence of self-harm among adolescents aged 10-14 years were Western Sub-Saharan Africa (14.1%), Eastern Sub-Saharan Africa (13.1%), and Andean Latin America (12.2%). Among adolescents aged 15-19 years, the regions with the highest prevalence were Andean Latin America (41.3%), Oceania (41.3%), and North Africa and the Middle East (41.2%). For the 20-24-year age group, the prevalence of self-harm was the highest globally and across the 21 super-regions in both 1990 and 2021.

**Figure 3:**
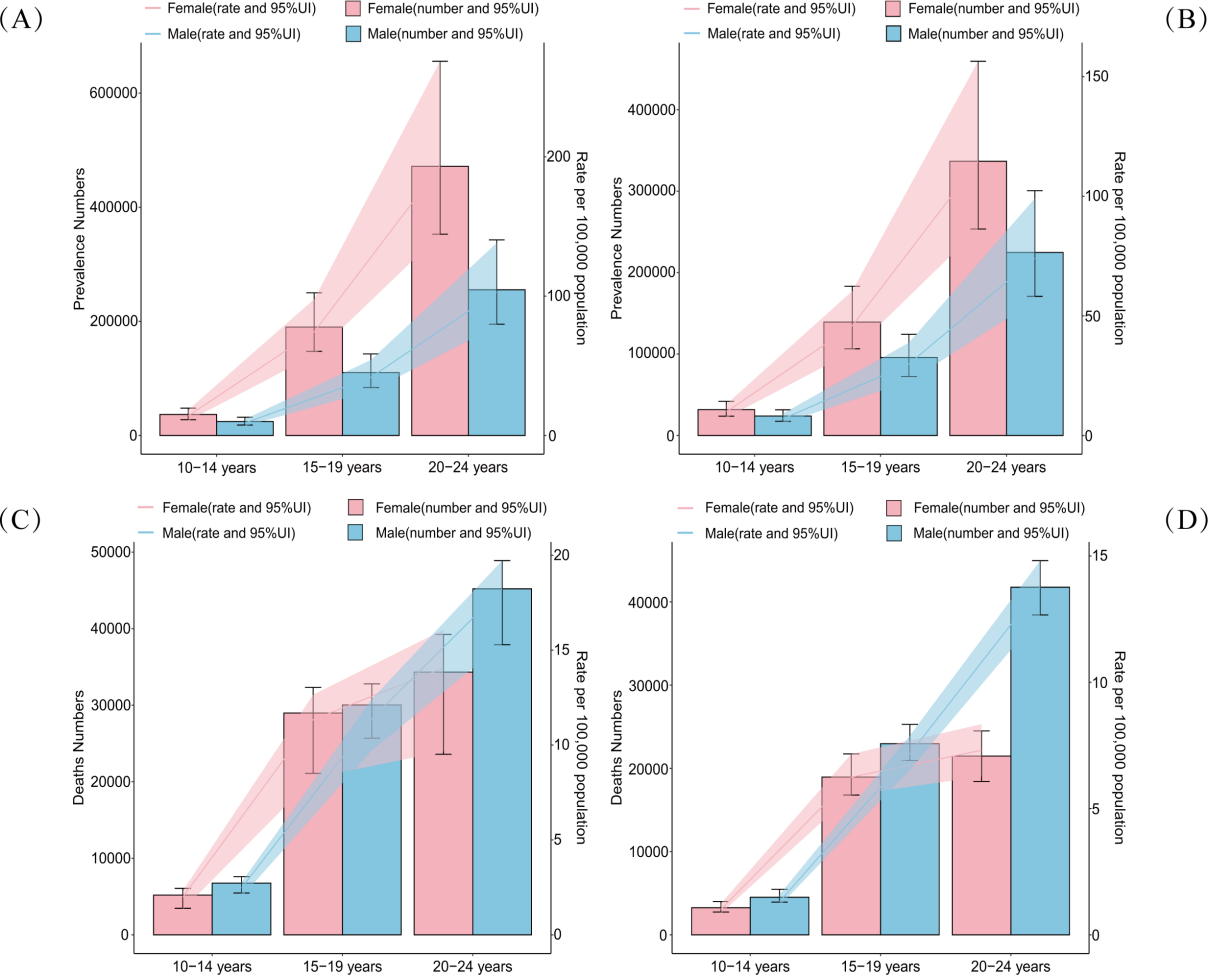
Double Y-axis plots of age-gender-time trends in prevalence rate (numbers) and deaths rate (numbers) of self-harm among adolescents in 1990 [**(A, B)** and 2021 **(C, D)**]. The left Y-axis represents the number in units of 100,000 and the right Y-axis represents the rate per 100,000 population. Blue represents data from male adolescents, and red represents data from female adolescents. UI: Uncertainty Interval.

Regarding mortality, the burden of self-harm among adolescents also increased with age. Notably, the gender trend in mortality exhibited an opposite pattern to that of prevalence. While female adolescents had a significantly higher prevalence of self-harm than males, male adolescents experienced significantly higher self-harm-related mortality rates. Globally, adolescent self-harm mortality showed an overall downward trend in 2021 compared to 1990. However, among adolescents aged 20-24 years, the gender differences in self-harm mortality were more pronounced (**Figure 3**, and **Supplementary Table 2**).

### 3.3 Analysis of health inequalities by gender and region

**Figure 4:**
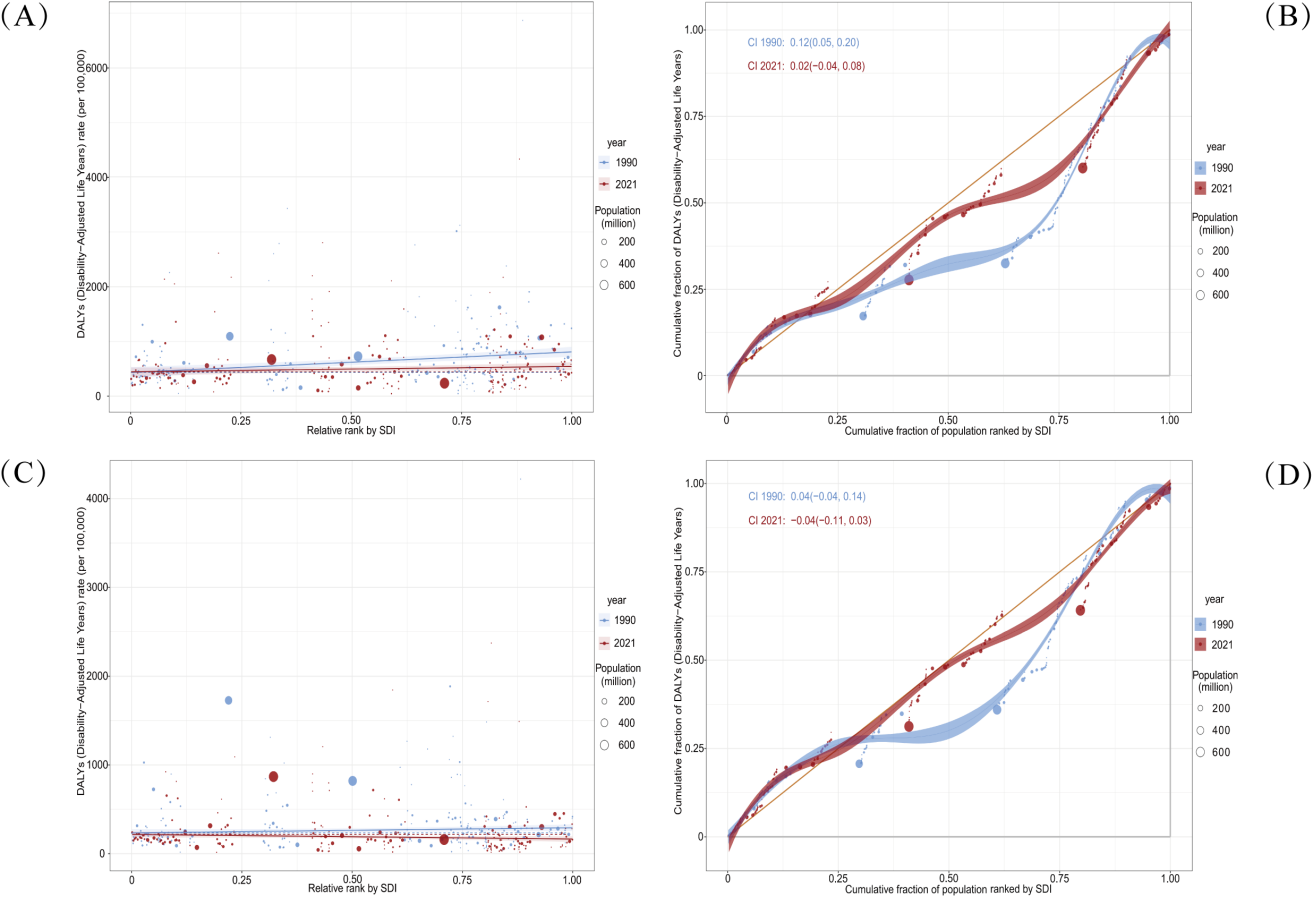
Health inequality regression curves and concentration curves for the DALYs of self-harm among adolescents based on gender worldwide, 1990 and 2021. **(A, B)** illustrate the SII and the concentration index for male adolescents. **(C, D)** illustrate the SII and the concentration index for female adolescents. The slope index of inequality depicts the relationship between SDI and age-standardized DALYs rates for each condition, with points representing individual countries and territories sized by population. The concentration index quantifies relative inequalities by integrating the area under the Lorenz curve, aligning DALYs distribution with population distribution by SDI. Blue represents data from 1990, and red represents data from 2021. DALYs, Disability-Adjusted Life-Years; SII, Slope Index of Inequality; SDI, Socio-Demographic Index.

Despite a reduction in the overall burden of self-harm among adolescents in 2021, gender- and region-based inequalities remained substantial. Significant absolute and relative inequalities were observed in relation to the SDI, with countries and regions of Middle and High SDI carrying a disproportionately higher burden. For male adolescents, the SII indicated that the difference in DALYs between countries and regions with the highest and lowest SDI narrowed from 378.34 (95% CI: 207.89, 548.80) in 1990 to 102.27 (95% CI: -34.39, 238.93) in 2021 (**Figure 4**). The concentration index shifted from 0.12 (95% CI: 0.05, 0.20) in 1990 to 0.02 (95% CI: -0.04, 0.08) in 2021 (**Figure 4**). For female adolescents, the SII narrowed from 57.93 (95% CI: -11.98, 127.84) in 1990 to -52.30 (95% CI: -106.14, 1.54) in 2021 (**Figure 4**). Meanwhile, the concentration index decreased from 0.04 (95% CI: -0.04, 0.14) in 1990 to -0.04 (95% CI: -0.11, 0.03) in 2021 (**Figure 4**). These findings suggest that while absolute health inequalities in the adolescent self-harm burden decreased from 1990 to 2021, the burden among male adolescents remained concentrated in Middle and High SDI countries and regions. In contrast, the burden among female adolescents became increasingly concentrated in Low SDI countries and regions. Relative inequality remained concentrated in Middle and High SDI countries and regions but showed an overall decline over time.

### 3.4 The effect of age-period-cohort on adolescent self-harm prevalence

**Figure 5:**
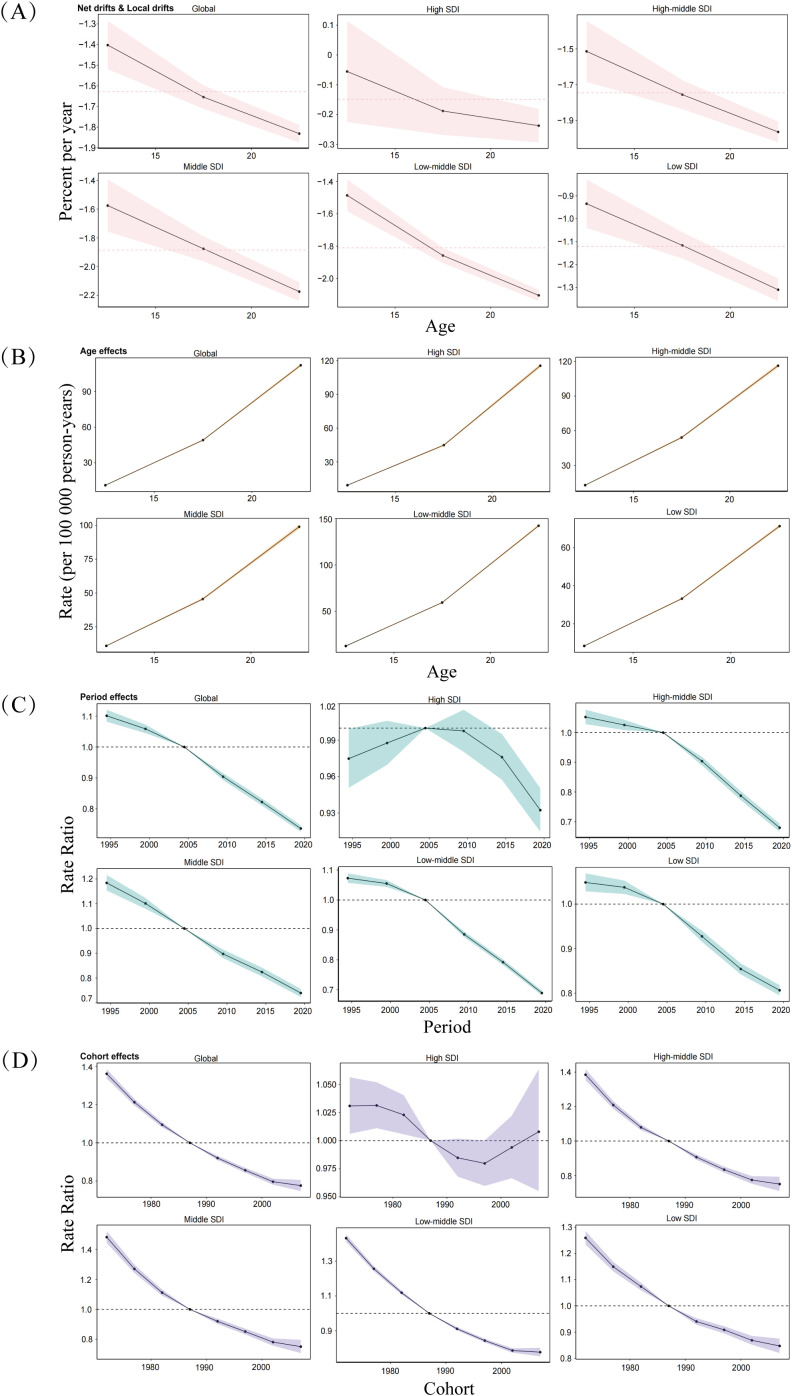
Age, period, and birth cohort effects on adolescent self-harm prevalence across SDI quintiles. **(A)** Local drift of prevalence from 1990 to 2021 for adolescent self-harm for three age groups (10-14, 15-19, 20-24 years). The dots and shaded areas denote the local drift (ie, APC of age-specific prevalence, % per year) and their corresponding 95% CI. **(B)** Age effects, **(C)** Period effects, and **(D)** Birth cohort effects are illustrated by the prevalence rate ratio. The dots and shaded areas denote the prevalence rates or rate ratios and their corresponding 95% CI. SDI: Socio-Demographic Index.

**Figure 5** illustrates the local drift calculated by the APC model, representing the AAPC in prevalence for each age group. A consistent decline in prevalence was observed across all age groups globally and in the five SDI regions. The most pronounced decline occurred among adolescents aged 20-24 years in the Middle and Low-middle SDI regions, with local drift coefficients of -2.17 and -2.10, respectively.

Analysis of age effects revealed significant differences in prevalence across age groups (**Figure 5**). The prevalence of self-harm increased with age, indicating that older adolescents were at a higher risk. This age effect was most pronounced in Low-middle SDI regions. Period effects analysis showed that the prevalence of self-harm among adolescents fluctuated more significantly in High SDI regions between 1990 and 2021, with an upward trend followed by a decline (**Figure 5**). However, the overall trend demonstrated a gradual decline in prevalence over time globally and across other SDI regions. Notably, no statistically significant difference in period effects was observed among Middle SDI regions (χ^2^ = 7.37, *P* = 0.12). Cohort effects analysis indicated a downward trend in the prevalence of self-harm among adolescents across different birth cohorts. However, the relative risk of self-harm was higher among earlier birth cohorts of adolescents. In High SDI regions, the relative risk of self-harm exhibited a declining trend followed by an increase, particularly among the most recent adolescent birth cohorts (**Figure 5**). These findings highlight the complex interplay of age, period, and cohort factors in shaping the prevalence of self-harm among adolescents globally and within different SDI regions.

### 3.5 The relationship between SDI and the burden of self-harm in adolescents

**Figure 6:**
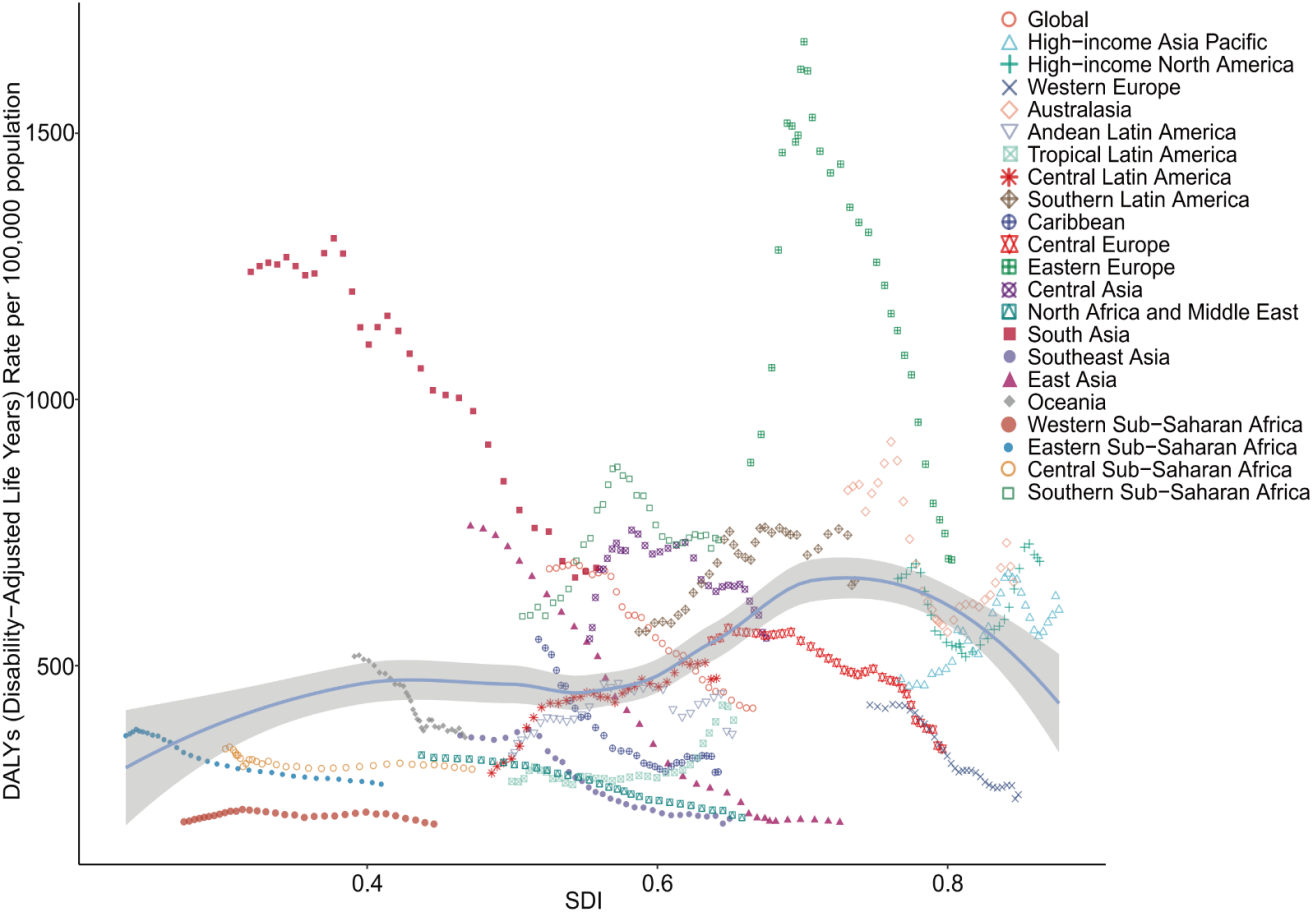
Non-linear relationships and trends between SDI and DALYs rates of self-harm among adolescents in 21 super-regions and globally from 1990 to 2021. DALYs, Disability-Adjusted Life-Years; SDI, Socio-Demographic Index.

Globally and across the 21 GBD regions, a nonlinear relationship was observed between the SDI and the age-standardized DALYs for adolescent self-harm (*r* = 0.324, *P* < 0.001). When the SDI was below 0.4, the burden of self-harm among adolescents increased gradually as the SDI rose, followed by a slight decrease in the range of 0.4-0.55. However, when the SDI was between 0.55 and 0.7, the burden of adolescent self-harm increased sharply with rising SDI. In contrast, when the SDI exceeded 0.7, the burden of self-harm among adolescents decreased rapidly as the SDI rose. Among regions, South Asia and East Asia demonstrated the most significant decreases in the burden of disease, whereas other regions exhibited either smaller decreases or slight increases. Notably, the Central Latin America region experienced the largest increase in burden, while the Eastern Europe and Southern Sub-Saharan Africa regions displayed a distinct pattern of an initial increase followed by a decrease (**Figure 6**, **Supplementary Tables 3**, **4**).

**Figure 7:**
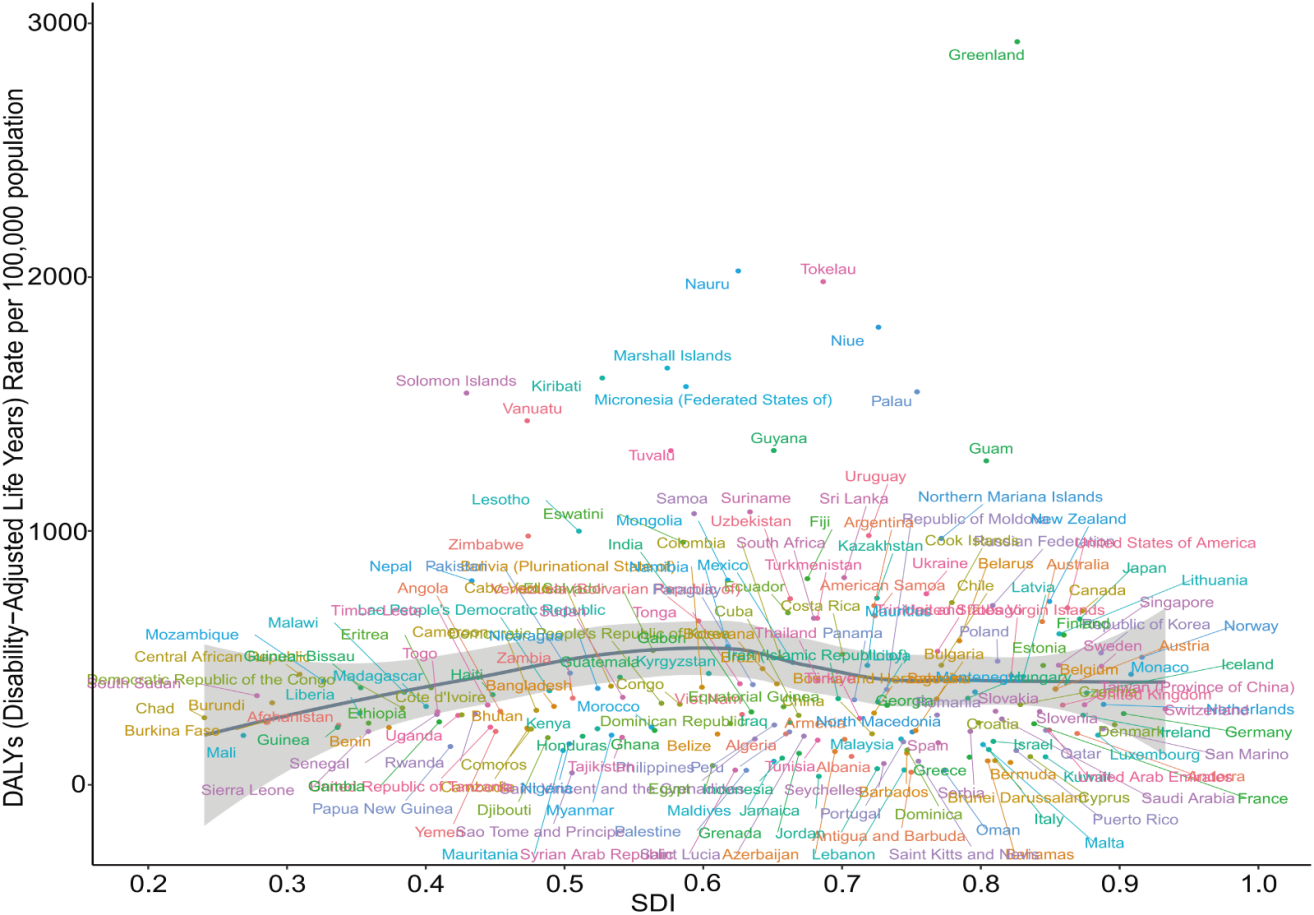
Non-linear relationship between SDI and DALYs in self-harm among adolescents in 204 countries and territories in 2021. DALYs, Disability-Adjusted Life-Years; SDI, Socio-Demographic Index.

In 2021, a similar nonlinear relationship between SDI and the age-standardized DALYs for adolescent self-harm was observed across 204 countries, though the pattern was less consistent. When the SDI was below 0.6, the burden of self-harm among adolescents increased as the SDI rose. However, when the SDI exceeded 0.8, the burden began to decline with increasing SDI, although this trend was not statistically significant (*r* = 0.024, *P* = 0.734) (**Figure 7**, **Supplementary Table 5**).

### 3.6 Joinpoint regression and BAPC predictive analysis results

**Figure 8:**
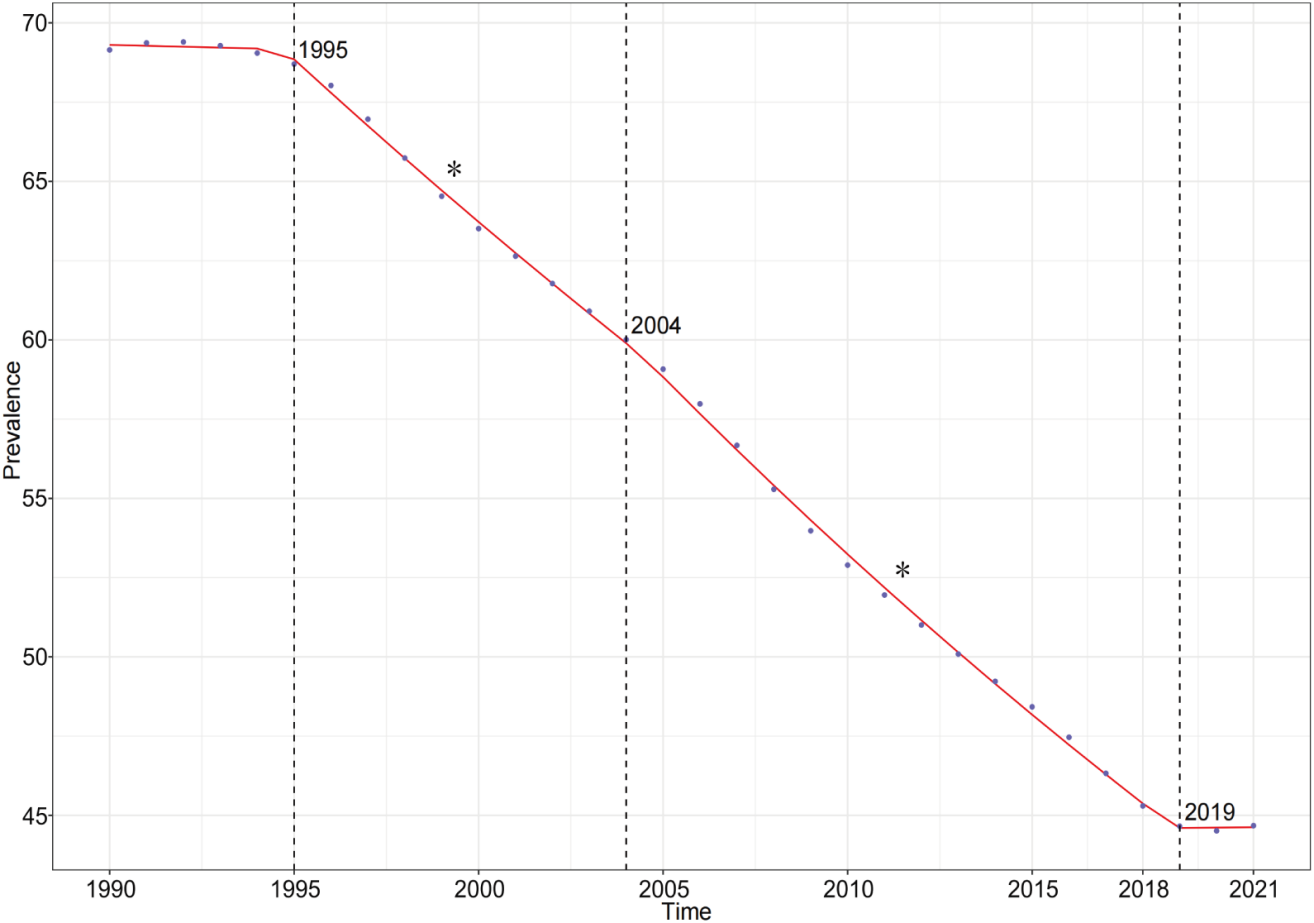
Joinpoint regression analysis results of prevalence from 1990 to 2021.* represents statistical significance *p* < 0.05.

The Joinpoint regression analysis revealed a general downward trend in the prevalence of self-harm among adolescents globally from 1990 to 2021. Specifically, the AAPC for the prevalence of adolescent self-harm was -0.799 (95% CI: -0.818, -0.780). Within this overall trend, three key Joinpoints were identified in 1995, 2004, and 2019. Prevalence showed no statistically significant change from 1990 to 1995 (APC: -0.042 (95% CI: -0.262, 0.179)), declined significantly from 1995 to 2004 (APC: -1.537 (95% CI: -1.612, -1.461)) and 2004 to 2019 (APC: -1.978 (95% CI: -2.023, -1.932)), and then showed no statistically significant change from 2019 to 2021 (APC: -0.021 (95% CI: -0.471, 0.516)) (**Figure 8**).

**Figure 9:**
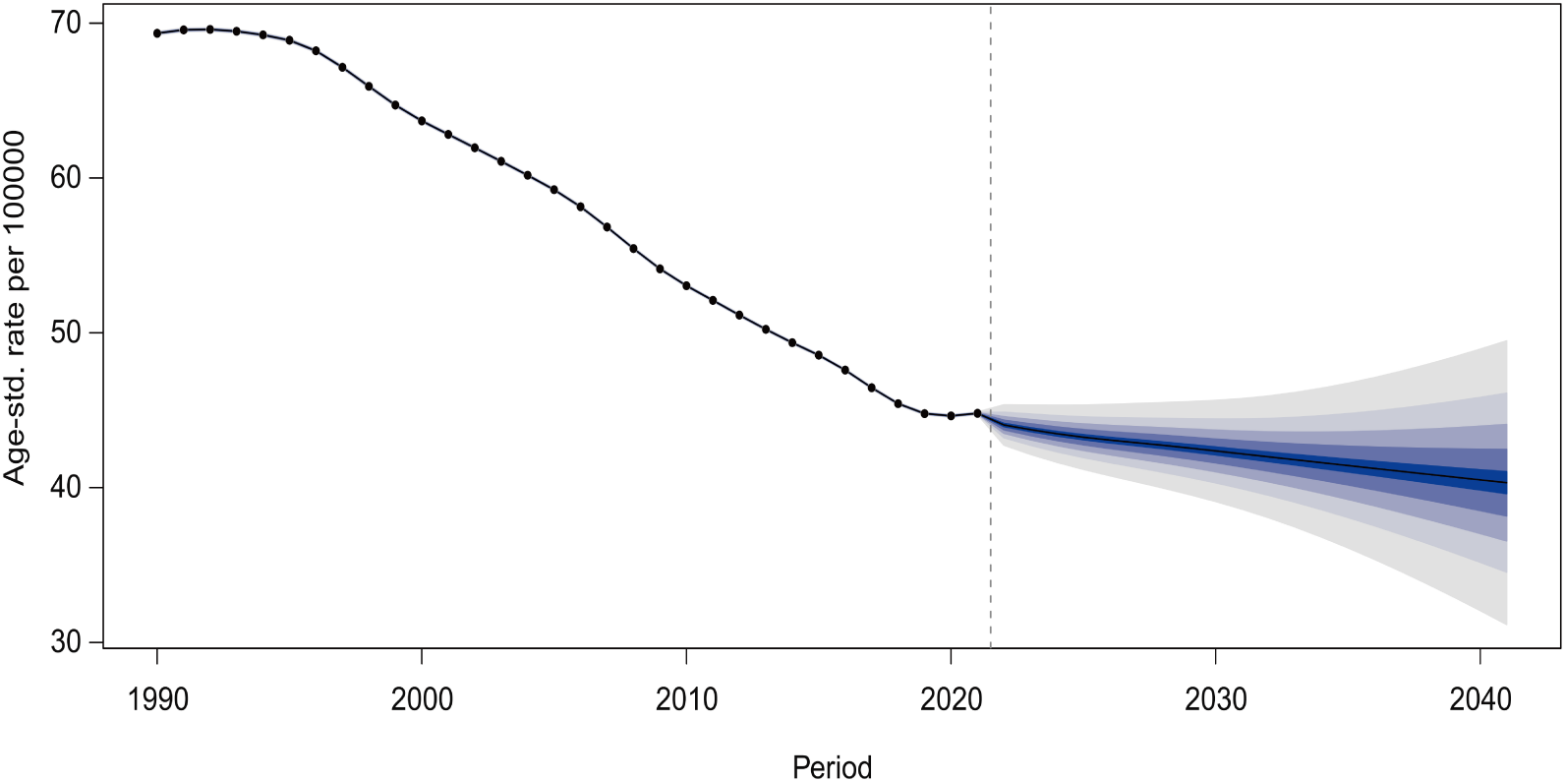
Projected changes in global self-harm prevalence among adolescents from 2022 to 2041.

Projections based on the BAPC model estimate that the global age-standardized prevalence of self-harm among adolescents will decline to 40.32 cases per 100,000 (95% CI: 29.41-51.23) by 2041 (**Figure 9**). Furthermore, projections of prevalence stratified by adolescent age groups indicate a gradual overall decline, with the most pronounced decrease observed in the population aged 20-24 years. Detailed projections of overall and age-specific age-based prevalence rates of self-harm among adolescents globally from 2022 to 2041 are provided in **Supplementary Tables 6**, **7**.

## 4 Discussion

Utilizing data from the GBD 2021 database, this study systematically analyzes the dynamic trends in the burden of self-harm among adolescents (aged 10-24 years) across five SDI regions, 21 GBD regions, and 204 countries and territories from 1990 to 2021. This study adopts an innovative approach by leveraging GBD 2021 data to explore and characterize age-period-cohort effects, gender-time-space trends, burden, and health inequalities associated with adolescent self-harm. Furthermore, it prospectively projects trends through 2041. Key time points of significant prevalence changes were identified, along with an assessment of the underlying mechanisms through which socioeconomic development interacts with the burden of self-harm. In line with the latest recommendations, the study focuses on adolescents aged 10-24 years, as this broader definition captures the biological, social, and neurocognitive development of this population. This contemporary framework has important implications for global public health. The study’s findings reveal that despite an overall global decline in the burden of self-harm (as indicated by ASIR, ASMR, ASDR, and EAPC metrics), substantial health inequalities persist between regions at differing levels of development and between genders. Notably, since 1990, the prevalence of self-harm in High SDI regions has stabilized or even slightly increased, with Southern Latin America experiencing the most significant rise. In contrast, the age effect is most pronounced in Low and Middle SDI regions, while period and cohort effects fluctuate more prominently in High SDI regions. Additionally, gender disparities are significant: female adolescents exhibit higher prevalence rates of self-harm than males, while males experience higher mortality rates, particularly in Middle and High SDI countries and regions. This study applies advanced analytical methods to provide fresh perspectives on the global burden of adolescent self-harm. These findings lay a crucial foundation for targeted public health policies, particularly in reducing gender and regional health inequalities, optimizing healthcare resource allocation, and enhancing mental health education.

The overall downward trend in the global burden of adolescent self-harm from 1990 to 2021 is likely linked to increased global awareness of mental health issues and the implementation of targeted interventions in recent years (36, 37). For example, high-income countries have generally established robust mental health support systems, promoted school-based mental health education, and, in some cases, introduced laws and policies aimed at reducing youth suicide and self-harm behaviors. Global initiatives such as the United Nations Sustainable Development Goals (SDGs) and the WHO Mental Health Action Plan 2013-2030, which aim to reduce suicide rates by one-third by 2030, also contribute to this decline (22). However, despite this overall decline, self-harm prevalence in High SDI regions has rebounded in recent years, showing a stable or slightly upward trend, particularly from 2019 to 2021. This phenomenon may be attributed to the exacerbation of adolescent mental health challenges during the COVID-19 pandemic. The COVID-19 pandemic has profoundly affected adolescent mental health, exacerbating stressors that contribute to self-harm behaviors. Increased social isolation, disruptions in education, economic instability, and limited access to mental health services have led to a concerning rise in psychological distress among young people (36). Studies have shown that lockdown measures and school closures significantly reduced social support networks, increasing the risk of loneliness, anxiety, and depression—all of which are strongly correlated with self-harm (38, 39). Additionally, disparities in digital access to mental health care further exacerbated inequalities, as adolescents in lower-income settings faced greater barriers to obtaining remote psychological support. Given these trends, the long-term mental health consequences of the pandemic warrant close monitoring, as they may contribute to sustained or even worsening self-harm prevalence in some regions beyond 2021.

Further analysis indicates that Southern Latin America experienced the largest increase in the burden of self-harm, followed by the Eastern European region. The high burden of self-harm in Southern Latin America and Eastern Europe may be attributed to several factors. In Southern Latin America, socioeconomic inequality, cultural taboos, ethnic disparities, and inadequate mental health service coverage for adolescents may contribute to underreporting and delayed intervention, exacerbating self-harm prevalence (40). In Eastern Europe, historical socio-political instability, high alcohol consumption rates, and limited mental health infrastructure have been linked to elevated suicide and self-harm rates among adolescents (41). Conversely, East Asia saw the largest decrease in the burden of self-harm, which may be attributed to effective policy interventions, cultural attitudes toward mental health, school-based mental health initiatives, and improved healthcare resources associated with economic development in the region (42).

Our findings reveal striking gender disparities in the global burden of adolescent self-harm. Female adolescents exhibit significantly higher prevalence rates than males, while males experience significantly higher mortality rates. These results align with previous studies and underscore the critical role of gender in the mechanisms underlying self-harm behavior (40). Among the gender differences that emerge from early adolescence, self-harm-related suicide rates tend to be 2-4 times higher in males, and 3-9 times higher in females for attempted suicide (43, 44). Research suggests that females are more likely to express emotional distress through non-lethal self-harm, while males are more likely to engage in more lethal methods, resulting in higher mortality rates (45, 46). Gender disparities also vary across levels of development. Inequalities in the burden of self-harm among male adolescents are more pronounced in Middle and High SDI regions, while inequalities among female adolescents are more prominent in Low SDI regions. This disparity may reflect the influence of socio-cultural norms, gender role expectations, and healthcare resource allocation. In Low SDI countries, female adolescents’ mental health needs may be underestimated or neglected due to their disadvantaged societal status and limited access to mental health services (47, 48). Conversely, male adolescents in Middle and High SDI countries may face greater social and economic pressures, coupled with cultural norms that discourage males from seeking mental health support, contributing to their higher mortality rates (48). The observed gender disparities in the prevalence and mortality of self-harm may be partially explained by psychosocial theories such as gender role stress and coping mechanisms. Males, particularly in High and Middle SDI regions, often face societal expectations of emotional resilience and self-reliance, which may discourage help-seeking behaviors and increase engagement in more lethal self-harm methods (49). In contrast, females tend to exhibit higher rates of non-lethal self-harm, potentially as a coping mechanism to express emotional distress (45, 46). Addressing these gender-specific barriers through targeted interventions, such as gender-sensitive counseling approaches and awareness campaigns, may be key to reducing self-harm mortality rates among male adolescents.

Analysis of age effects reveals that the burden of self-harm is highest among adolescents aged 15–24 years. This period represents a time of heightened vulnerability due to physiological and psychological changes, transitions in social roles, and increased exposure to external stressors. These findings emphasize the crucial importance of mental health interventions tailored to the specific needs of adolescents during this critical developmental stage (47). The analysis of period effects highlights the profound impact of historical and social contexts on the burden of adolescent self-harm. Period effects fluctuated more markedly in High SDI regions, where rapid societal changes, such as economic crises or global events like the COVID-19 pandemic, have significantly influenced adolescent mental health. In contrast, period effects in Low and Middle SDI regions showed a more stable downward trend, potentially reflecting slower societal transitions and limited mental health resources. Cohort analyses further reveal the influence of unique sociocultural and historical contexts experienced by adolescents of different birth years. For example, adolescents born during times of economic prosperity in High SDI regions may face fewer socioeconomic stressors, while those born during economic downturns may experience greater psychological burdens (48). Additionally, earlier birth cohorts were found to have a higher risk of self-harm compared to more recent cohorts, possibly due to improvements in mental health awareness, interventions, and healthcare access over time. These findings suggest that cohort-specific interventions, informed by the sociocultural and historical experiences of different generations, could be a critical direction for reducing the burden of self-harm in the future.

The observed nonlinear relationship between SDI and ASDR highlights the complex interaction between socioeconomic development and the burden of self-harm. While socioeconomic development generally correlates with reductions in self-harm burden, this relationship is not linear. High SDI countries and regions often exhibit a paradoxical increase in the burden of self-harm, a phenomenon known as the “mental health paradox of modernization.” This paradox suggests that social pressures, cultural shifts, and changes in family dynamics associated with rapid economic development may exacerbate mental health challenges (50). In contrast, Low SDI countries face a slower decline in the burden of self-harm due to limited healthcare resources, incomplete data collection, and inadequate mental health education. High SDI countries, despite having sufficient healthcare resources, have experienced a rebound in self-harm burden due to rising social competition, individualism, and social isolation (51). These findings underscore that economic development alone is insufficient to address the burden of self-harm among adolescents. Strengthening mental health services, optimizing healthcare resource allocation, and enhancing social support systems are critical to effectively reducing the global burden of self-harm.

The most notable innovation of this study lies in its integration of advanced statistical methods, including APC modeling, BAPC predictive modeling, Joinpoint regression, and health inequality analyses. Compared to traditional cross-sectional studies, this comprehensive approach captures the complex developmental trajectory of the adolescent self-harm burden in a dynamic and multidimensional manner. Additionally, the study’s use of a broad definition of adolescents (aged 10-24 years) allows for a more age-appropriate understanding of self-harm and uncovers intersecting patterns of gender and geographic inequalities, thus expanding traditional epidemiological perspectives on adolescent self-harm.

Despite these strengths, the study has several limitations. First, self-harm data from low-income countries in the GBD 2021 database may suffer from underreporting or inaccuracies, potentially leading to under- or overestimations of the results (52). Future studies should incorporate statistical weighting methods to improve the accuracy of estimates and advocate for enhanced data collection and reporting in low-income regions. Second, forecasting models such as BAPC rely on the assumption that socioeconomic development and policy interventions will follow current trends, which may overlook the potential impact of unforeseen global events or disruptions. Future research should explore more flexible and dynamic modeling approaches to better account for complex environmental changes.

Finally, this study did not fully explore the deeper mechanisms underlying gender differences in the burden of self-harm. For example, higher self-harm mortality rates among males in High SDI regions may be influenced by societal gender norms that discourage emotional expression and help-seeking behavior, but this hypothesis was not thoroughly tested. Similarly, the specific manifestations of gender differences in self-harm across varying psychological and sociocultural contexts were not adequately compared. Future research should integrate social, psychological, and biological factors into a multilevel analytical framework to comprehensively uncover the mechanisms driving self-harm behaviors among adolescents. Additionally, future studies should develop and evaluate more individualized intervention strategies, tailored to different groups of adolescents based on gender, age, and regional characteristics. For instance, interventions for female adolescents should focus on addressing emotional distress and improving access to mental health services, especially in Low SDI regions. For male adolescents, particularly in Middle and High SDI regions, efforts should aim to reduce stigma surrounding mental health and encourage help-seeking behaviors, as well as address societal pressures and expectations that contribute to their higher mortality rates.

In conclusion, this study provides a comprehensive analysis of the global burden of self-harm among adolescents, highlighting the complex interplay of age, period, cohort, gender, health inequalities, and socioeconomic factors. The findings underscore the urgent need for targeted interventions that address the specific needs of different adolescent populations, particularly in the context of ongoing global changes and persistent health inequalities. By integrating advanced statistical methods and adopting a broader definition of adolescence, this research contributes to a more nuanced understanding of self-harm and provides a valuable foundation for developing effective public health policies and interventions. Policy interventions play a crucial role in shaping future trends in adolescent self-harm. School-based mental health programs have proven effective in increasing awareness, reducing stigma, and providing early intervention for at-risk adolescents (53, 54). Suicide prevention hotlines and crisis intervention services have also demonstrated significant benefits in reducing self-harm behaviors, particularly when widely accessible through digital platforms (55, 56). Moreover, the rise of digital mental health interventions, including app-based cognitive behavioral therapy (CBT) and online peer support networks, presents a promising avenue for improving mental health access, particularly in underserved regions (56). Moving forward, integrating evidence-based mental health programs into national education and healthcare systems will be essential in sustaining the global decline in adolescent self-harm rates.

## 5 Conclusion

Over the past three decades, the global burden of adolescent self-harm has shown a consistent decline. However, significant disparities persist across gender, geographic regions, and SDI. Addressing these inequalities remains a critical priority for future research and intervention. In High SDI regions, targeted psychological support for male adolescents is essential, while in Low SDI regions, greater attention must be given to the mental health needs of female adolescents. Equitable allocation of healthcare resources is also crucial, particularly in enhancing medical and technical support in resource-limited settings.

To effectively mitigate adolescent self-harm, a multifaceted approach is required. Strengthening school-based mental health services, expanding community outreach programs, and reducing barriers to professional psychological care are key strategies. The integration of mental health education into school curricula and the training of teachers and counselors to identify and support at-risk students are particularly important. Additionally, digital mental health interventions, including telehealth services and online support platforms, can help bridge gaps in care, especially in underserved regions. Gender-sensitive strategies should be prioritized to ensure interventions are tailored to the distinct psychological needs of male and female adolescents.

Future research should focus on longitudinal studies to assess the sustained impact of digital mental health interventions in preventing self-harm. Given the increasing reliance on digital therapy platforms and online peer support networks, evaluating their long-term effectiveness is imperative. Additionally, further investigation is needed to understand the prolonged psychological effects of the COVID-19 pandemic, particularly among vulnerable subpopulations. A rigorous evaluation and refinement of existing policies, coupled with the development of innovative, data-driven interventions, will be essential in preventing a potential resurgence of self-harm behaviors. Given the substantial heterogeneity in burden, trends, and inequalities, future interventions must be context-specific, culturally sensitive, and implemented through a gender-responsive framework. By adopting comprehensive, evidence-based strategies, policymakers can play a pivotal role in reducing the global burden of adolescent self-harm.

## Data Availability

The original contributions presented in the study are included in the article/**Supplementary Material**. Further inquiries can be directed to the corresponding author.
